# Alpha EEG Activity and Pupil Diameter Coupling during Inactive Wakefulness in Humans

**DOI:** 10.1523/ENEURO.0060-21.2022

**Published:** 2022-04-13

**Authors:** Rodrigo Montefusco-Siegmund, Miriam Schwalm, Eduardo Rosales Jubal, Christ Devia, José I. Egaña, Pedro E. Maldonado

**Affiliations:** 1 Instituto de Aparato Locomotor y Rehabilitación, Human Cognitive Neurophysiology and Behaviour Laboratory, Facultad de Medicina, Universidad Austral de Chile, Valdivia, 5111815, Chile; 2 Department of Biological Engineering, Massachusetts Institute of Technology, Cambridge, Massachusetts 02139; 3 Competence Center for Methodology and Statistics, Luxembourg Institute of Health, Strassen 1445, Luxembourg; 4 Biomedical Neuroscience Institute, Facultad de Medicina, Universidad de Chile, Santiago, 8380000, Chile; 5 Departamento de Neurociencias, Facultad de Medicina, Universidad de Chile, Santiago, 8380000, Chile; 6 Departamento de Anestesiología y Medicina Perioperatoria, Facultad de Medicina, Universidad de Chile, Santiago, 8380456, Chile

**Keywords:** arousal, cortical states, neural oscillations, resting state

## Abstract

Variations in human behavior correspond to the adaptation of the nervous system to different internal and environmental demands. Attention, a cognitive process for weighing environmental demands, changes over time. Pupillary activity, which is affected by fluctuating levels of cognitive processing, appears to identify neural dynamics that relate to different states of attention. In mice, for example, pupil dynamics directly correlate with brain state fluctuations. Although, in humans, alpha-band activity is associated with inhibitory processes in cortical networks during visual processing, and its amplitude is modulated by attention, conclusive evidence linking this narrowband activity to pupil changes in time remains sparse. We hypothesize that, as alpha activity and pupil diameter indicate attentional variations over time, these two measures should be comodulated. In this work, we recorded the electroencephalographic (EEG) and pupillary activity of 16 human subjects who had their eyes fixed on a gray screen for 1 min. Our study revealed that the alpha-band amplitude and the high-frequency component of the pupil diameter covariate spontaneously. Specifically, the maximum alpha-band amplitude was observed to occur ∼300 ms before the peak of the pupil diameter. In contrast, the minimum alpha-band amplitude was noted to occur ∼350 ms before the trough of the pupil diameter. The consistent temporal coincidence of these two measurements strongly suggests that the subject’s state of attention, as indicated by the EEG alpha amplitude, is changing moment to moment and can be monitored by measuring EEG together with the diameter pupil.

## Significance Statement

Attention is a cognitive process through which an organism selects information based on adaptive behavior. Alpha-band EEG activity and pupil dynamics account for the variations in attention during cognitive tasks. During natural behavior, it is challenging to assess modulations of attention over time solely through EEG signals. Therefore, we explored whether pupillary activity could reflect similar processes. We measured the spontaneous fluctuations of EEG alpha-band activity and pupil diameter and found a robust temporal relationship between the two, suggesting fluctuations in attention.

## Introduction

The behavioral responses of animals to environmental demands vary from second to second. Psychophysical studies consistently show that we never respond in the same way to identical stimuli in the same fashion (Aton, 2013). Currently, the biological reasons for such variabilities in neuronal and behavioral responses are extensively studied. A parsimonious explanation postulates that the internal system is continuously changing, and so, however similar the external environment may be, those internal fluctuations affect the individual differently at each moment (Lee and Dan, 2012; McCormick et al., 2020). What components of these intrinsic variations directly affect the relationship of our body with the external environment? One particular focus has been placed on brain states and their relationship with behavior (McCormick et al., 2015; Samaha et al., 2020). As cognitive processes are associated with particular brain dynamics, changes in the global state of the brain can affect these dynamics and, consequently, behavior (Palva and Palva, 2007; Jensen et al., 2014). Changes in levels of attention, for example, are related to fluctuations in oscillatory activity at specific frequencies (Jensen et al., 2012; Gulbinaite et al., 2017). Human electroencephalography (EEG) data suggest that alpha oscillations play an essential role in shaping cortical states in sensory areas of the brain to optimize performance (Jensen and Mazaheri, 2010). Several visual perception studies have shown that anticipatory alpha activity reflects the orientation of attention (Foxe et al., 1998; Sauseng et al., 2005; Rihs et al., 2009; Cosmelli et al., 2011). Variation over time of alpha phase (Varela et al., 1981; Milton and Pleydell-Pearce, 2016), frequency (Samaha and Postle, 2015), and amplitude (Haegens et al., 2011; Händel et al., 2011) have been associated with behavioral performance.

Another especially interesting indicator of brain states is pupillary activity. In humans, pupil diameter is controlled by sympathetic innervation releasing noradrenaline (NA; for updated review, see Mathôt, 2020). The primary source of NA synthesis in the CNS is the locus coeruleus (LC). LC neurons innervate the radial muscle of the iris, which dilates the pupil and, concurrently, this NA release widely modulates cortical activity (Joshi et al., 2016; Joshi and Gold, 2020). In fact, variations in pupil diameter are not only related to changes in luminance, but also to higher-order cognitive processes such as visual awareness (Naber et al., 2011), covert attention (Binda and Murray, 2015), visual memory (Montefusco-Siegmund et al., 2017), emotional perception (Vasquez-Rosati et al., 2017) and working memory (Zokaei et al., 2019), among others (for review, see Mathôt, 2018). However, spontaneous variations in pupil diameter (i.e., variations not elicited by external input) may indicate variations in cognitive functions over time. For example, in a recent report, highly attentive moments, detected via task performance, exhibited larger pupil size, and a pupil-triggering procedure elicits differences in sustained attention, as indexed by response time (Keene et al., 2021). Evidence from animal studies shows that there is a close relationship between the state of brain activity and pupil dynamics. In rodents, during quiet wakefulness, spontaneous pupil dynamics are strongly linked to cortical (Poulet, 2014; Vinck et al., 2015; Reimer et al., 2016; Yüzgeç et al., 2018), hippocampal (McGinley et al., 2015), and subcortical (Pais-Roldán et al., 2020) activity. These results prompted the hypothesis that similar changes in the oscillatory profile of the human EEG can be captured through pupillometry (Schwalm and Jubal, 2017). There is increasing evidence in humans showing a clear relationship between neurovascular responses in early visual areas and the pupillary signal (Yellin et al., 2015; DiNuzzo et al., 2019; Mäki-Marttunen, 2021) supporting the possibility of pupillometry being linked to EEG signals. Indeed, a recent study showed a significant correlation between occipital alpha power and pupillary diameter during intertrial periods of rest (Ceh et al., 2020). However, evidence for a spontaneous relationship between alpha power and pupillary dynamics remains limited.

As different characteristics of alpha oscillation have an important relationship with individual performance (Händel et al., 2011; Jensen et al., 2012) and pupil diameter is a good predictor of behavioral outcome (Gelbard-Sagiv et al., 2018; Joshi and Gold, 2020), we hypothesized that, alpha activity and pupil diameter should covariate, suggesting variations in the level of attention over time. In the present study, we extend the limited evidence by studying spontaneous variations of alpha activity and pupil diameter in human subjects during inactive wakefulness and found a high correlation between the high-frequency component of pupil dynamics and the amplitude of alpha oscillation.

## Materials and Methods

### Subjects

A total of 17 subjects with normal or corrected-to-normal vision participated in this experiment. Of these, 5 were females, 13 were right handed, and 8 had right ocular dominance. The average age of the subjects was 31.41 ± 7.17 years. As stated in the Results section, one individual presented high levels of noise, and so only 16 subjects were considered for the analyses. All subjects were volunteers who had provided written informed consent to participate in the research; the Ethics Committee approved the consent form and all experimental protocols for research in humans of the Faculty of Medicine from the Universidad de Chile. All recordings were performed within a 32 d period.

### Task

The volunteers were instructed to maintain their gaze on a fixation cross in the middle of a plain gray screen ([173 173,173] RGB) for 1 min. Fixating on a point can be a strenuous task, which compounded with compelling evidence that longer periods of inactive wakefulness induce low-frequency oscillations of the pupil diameter called hippus (Bouma and Baghuis, 1971; Wilhelm et al., 1998; Turnbull et al., 2017), led us to limit our recordings to 1 min; thus seeking to preserve more physiological pupil dynamics. The subjects were seated in a dark room with their chins rested. The recordings were part of a previous study (Devia et al., 2017).

### Recordings

Whole-brain EEG was recorded with 32 electrodes (ActiveTwo, BioSemi), using the International 10–20 system layout. In addition, eight electrodes were used; one on each mastoid for rereferencing, and the remaining six recording the electrooculogram (EOG), placed above, below, and on the outer canthi of each eye. The EEG and EOG signals were recorded at 2048 Hz. Records were preamplified and referenced to CMS (Common Mode Sense) and DRL (Driven Right Leg) electrodes during acquisition, with the band filter between DC and 400 Hz, and store. The gaze position and pupil diameter were recorded using a chin-rest eye-tracking system at 500 Hz (EyeLink 1000, SR Research).

### Data analyses

**Figure 1.**
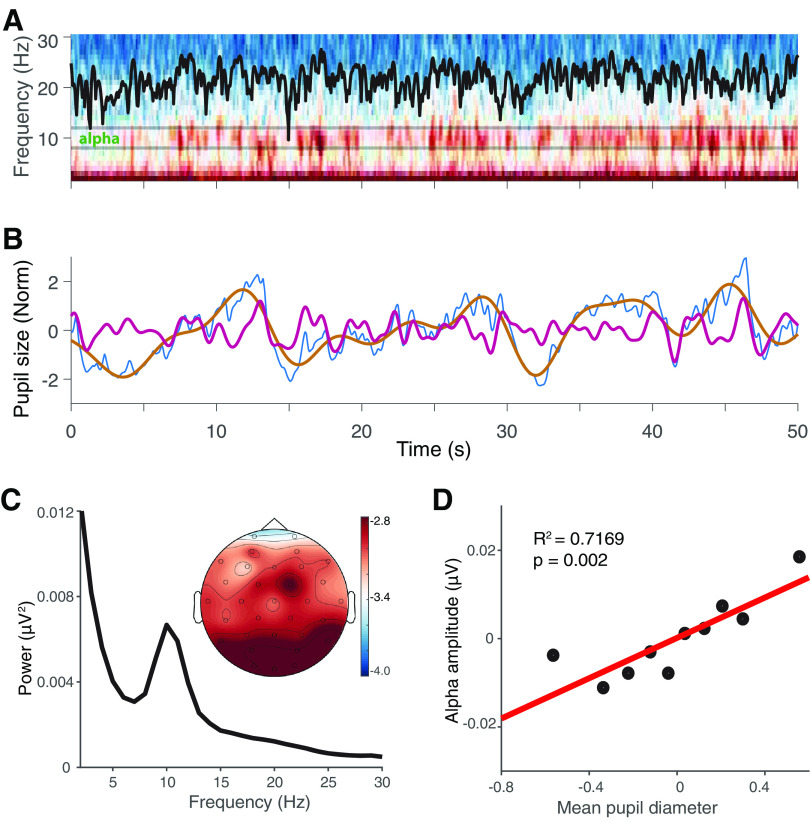
Pupil size and alpha-activity fluctuations are correlated. ***A***, Spectrogram of Oz channel during the whole duration of the gaze fixation period. Black parallel lines define the alpha band of an individual subject (subject 2). The mean normalized power in the alpha band is shown (black trace). ***B***, Normalized pupil diameter during fixation period. Raw pupil trace (blue), low-frequency pupil component (brown), and high-frequency pupil component (purple). ***C***, Grand average of power spectrum of parietal–occipital electrodes (*n* = 16 subjects). Inset, Topographical distribution of mean alpha power. ***D***, Mean amplitude of the high-frequency component of the pupil dynamic versus mean alpha power and the corresponding linear fit (red) averaged across all subjects (Extended Data Fig. 1-1).

**Figure 2.**
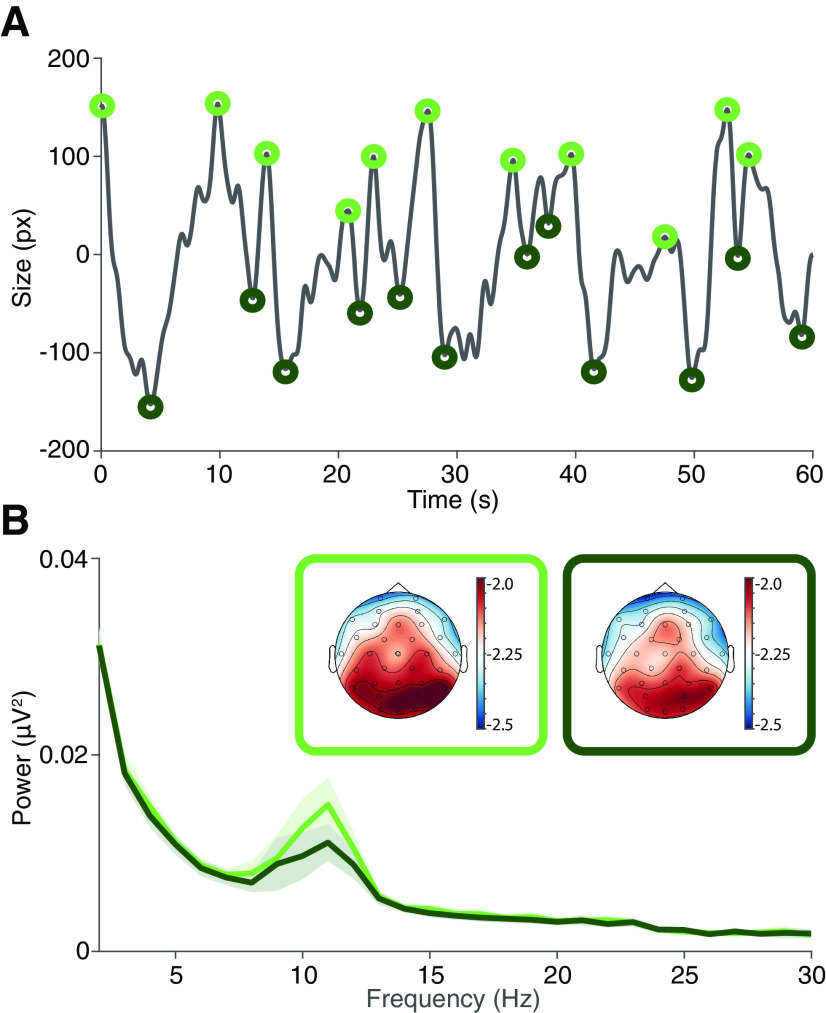
Pupil size peaks and troughs are associated with increases and decreases of alpha power. ***A***, High-frequency pupil diameter dynamic during the task. Pupil amplitude peaks (light green) and troughs (dark green). ***B***, Average power spectrum of parietal–occipital electrodes (see Materials and Methods) for epochs centered at peak (light green) and trough (dark green) of pupil diameter (*n* = 16; shadows correspond to SEM). Inset, Spatial distribution of the alpha band in the pupil peak (light green) and pupil trough (dark green) conditions.

**Figure 3.**
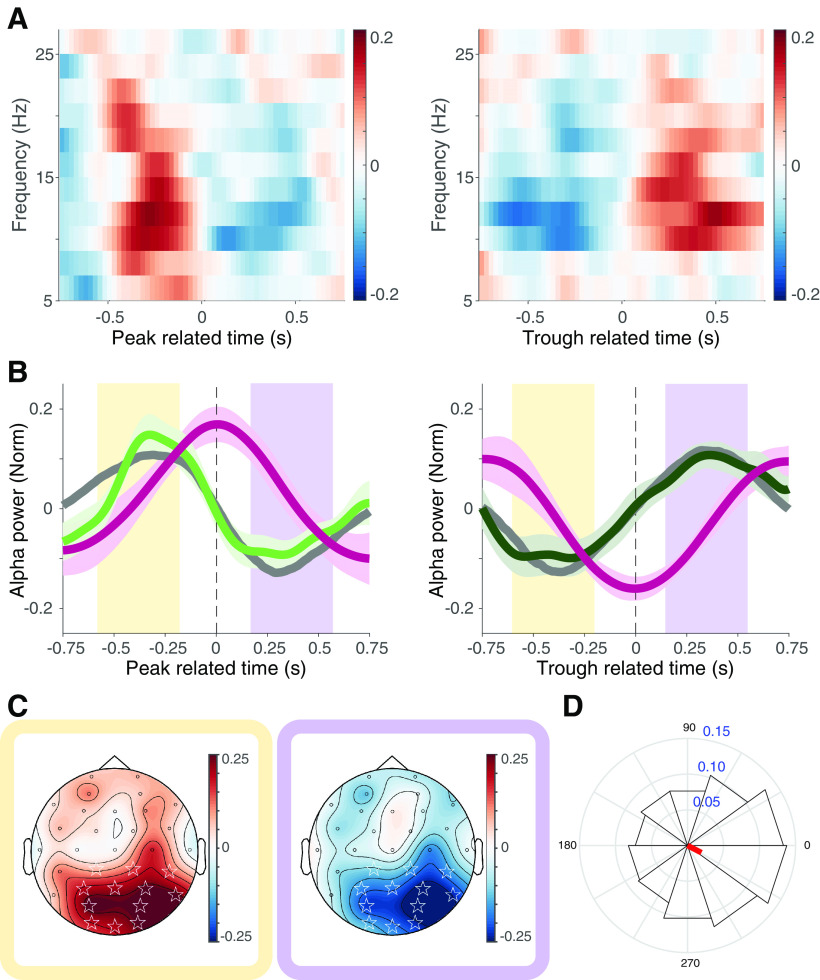
Alpha power is modulated around peaks and troughs in pupil diameter. ***A***, Time-resolved normalized power spectrum of the same electrodes in Figure 2*B* centered around the peak (left) and trough (right) of pupil diameter. Color bar represents the relative change in respect to the whole epoch. ***B***, Alpha power in time related to the peak (left) and trough (right) of pupil diameter. Normalized pupil size (red), first derivative (black), and center of the event (segmented line). Shadows represent SEM. ***C***, Spatial distribution of the difference in alpha band between peak and trough condition. Analysis windows were set from −0.6 to −0.2 s (left; ***B***, yellow shadow) and from 0.2 to 0.6 s (right; ***B***, purple shadow). *p* < 0.001 in a cluster-based permutation test (white stars). Color bar represents the relative change with respect to the whole epoch. ***D***, Polar histogram of alpha power in relation to high-frequency pupil phase. Red line represents the mean resultant vector across the whole trial of all subjects (Extended Data Fig. 3-1).

Pupil data were corrected to remove blinks and out-of-screen saccades that could cause loss of data. Windows where data loss occurred were replaced with linearly interpolating the missing values with the average of the previous 16 ms and the following 16 ms. Data were smoothed as implemented in the *smooth* function in MATLAB (release 2016b; MathWorks), using a 0.1% of total length window and applying a robust local regression using weighted linear least squares and a first-degree polynomial model. Subsequently pupil data from both eyes were averaged and normalized by referencing the whole recording and upsampled to 1000 Hz. Using a Brickwall frequency-domain filter of order 2, we obtained two bands of pupil signals. In this manner, the low-frequency band was defined as the pupil signal bandpass filtered between 0.05 and 0.2 Hz, and the high-frequency band was defined between 0.2 and 1 Hz. As highlighted before, we only reported the results for the high-frequency pupil diameter oscillations. Local peaks and troughs were detected based on local maxima and minima, separated by at least 3 s from previous or following smallest peaks and troughs. These peak and trough timepoints were used to epoch EEG data from 1 s before to 1 s after the respective events (see Fig. 2*A*). EEG analysis was performed in MATLAB using the FieldTrip toolbox (release 20171117). The recordings were rereferenced to the average of all EEG channels. We decomposed the data based on independent component analysis and visually compared the EOG channels with the identified independent components that had ocular content (blinks or saccades) and then reconstructed the signal. Depending on signal quality, between one and four independent components were rejected. Then, the data were decimated to a sampling rate of 1 kHz. Muscle, movement, and electrode artifacts were discarded through visual inspection. Spectrograms were calculated with a 500 ms moving window and a Hanning taper, followed by a Fourier transform. As we lacked a proper baseline period, in cases where normalization was applied (Figs. 1*A*, 2*C*), we used the whole epoch to calculate the relative change of power. The power spectrum in Figure 1*C* was calculated over the signal of the whole session, segmented in 1 s epochs using a Hanning taper, followed by a spectral decomposition using the Fourier transform. In the same way, the power spectrum from Figure 2*B* was calculated over 2 s epochs centered over pupil peaks and troughs. Continuous 8–12 Hz bandpass-filtered EEG and pupil data were segmented in 1 s epochs with 25% overlapping. We measured the mean pupil amplitude in each epoch and sorted the EEG according to pupil amplitude deciles. We calculated the corresponding mean amplitude of the alpha activity by using the Hilbert transform, as implemented in Fieldtrip (Fig. 1*D*). When alpha-band power was represented over time (Fig. 3*A*), we averaged the normalized power in the 8–12 Hz band from channels P3, Pz, PO3, O1, Oz, O2, PO4, and P4, as calculated in the time–frequency analysis. For the pupil phase–alpha amplitude coupling analysis, we concatenated the data of the whole session from all included subjects (*n* = 16), in vectors of high-frequency filtered pupil angles and alpha power. Subsequently, we calculated the mean alpha amplitude for each bin of the angles.

10.1523/ENEURO.0060-21.2022.f1-1 Figure 1-1 Individual correlation between pupil size and alpha amplitude. Left, Traces in light red correspond to each individual correlation between the pupil size deciles and the corresponding alpha amplitude at Oz location. Gray circles correspond to the averages of the decile used to calculate the correlations. Red line and black circles are the same averaged values shown in Figure 1*D*. Right, Black squares correspond to individual *r*^2^ and *p* value results from each individual correlation. Download Figure 1-1, TIF file.

10.1523/ENEURO.0060-21.2022.f3-1 Figure 3-1 Normalized cross-correlation between pupil size and alpha power. ***A***, Traces in gray correspond to each individual cross-correlation between the time course of the pupil size signal and the alpha power at occipital electrodes centered at peak pupil size. Green line represents the mean of all subjects (left). Same analysis as in left panel but centered at the trough of pupil size (right). ***B***, Same as ***A***, but for flipped EEG signal. Download Figure 3-1, TIF file.

### Statistics

To establish whether the power differences between peak and trough conditions were significantly different from 0, a group-based nonparametric randomization test was applied within subjects (Maris and Oostenveld, 2007). By grouping neighboring channels that showed the same effect, this test addresses the problem of multiple comparisons and takes data dependency into account. For each channel, a dependent sample *t* value was calculated. All samples for which the calculated *t* value exceeded an a priori threshold (uncorrected *p* < 0.05) were selected, and subsequently clustered based on spatial adjacency. The sum of the *t* values within a group was used as the group-level statistic. The group with the maximum sum was subsequently used as a test statistic. By randomizing the data between the two conditions and recalculating the test statistic 1000 times, we obtained a reference distribution of maximum cluster *t* values to evaluate the statistics of the real data. For circular-to-linear correlation analysis, we used the function *circ_corrcl* from the Circular Statistics Toolbox (Berens, 2009). The maximum values of the cross-correlation analyses between pupil diameter and alpha power were evaluated for statistical significance through a permutation test using the values of alpha power shuffled over time. For the control cross-correlation analyses, the pupil signals were reversed in time before the analysis described above, while the alpha-band signals remained unchanged.

## Results

### Occipital alpha amplitude and pupil diameter are correlated during inactive wakefulness

To determine whether pupil dynamics can account for different states of cortical activity, we studied the EEG signals in relation to pupil diameter variations during gaze fixation. During this period, EEG activity showed spontaneous fluctuations in different frequency bands, wherein the more distinctive feature was the modulation of alpha-band (8–12 Hz) power in time (Fig. 1*A*, black trace). Pupil fluctuations showed a high-frequency (0.2–1 Hz; Fig. 1*B*, red trace) and a low-frequency (0.05–0.2 Hz; Fig. 1*B*, brown trace) component (Reimer et al., 2016). Low-frequency pupil oscillations were observed to develop in time windows of seconds to minutes, which, in our paradigm, is not enough to perform proper estimation of its effects. Hence, we focused our analysis on the high-frequency component alone. The power spectrum in the posterior electrodes (P3, Pz, PO3, O1, Oz, O2, PO4, and P4), averaged over all subjects, was characterized by a prominent peak in the alpha band (Fig. 1*C*). The scalp topology of alpha power indicates that the dominating contributor of alpha power originates from electrodes in the visual/posterior area (Fig. 1*C*, inset). To explore the potential relationship between pupil size and alpha amplitude, we sorted the filtered pupil data in pupil diameter deciles and calculated their corresponding mean alpha amplitude averaged across all subjects. Alpha amplitude increased from −0.0038 μV (±0.0154 SD) in the first decile of pupil size to 0.0195 μV (±0.0263 SD) in the 10th decile, based on high-frequency bandpass-filtered pupil signal. Pearson correlation analysis showed a linear association of *r*^2^ = 0.7169 (*n* = 10, *p* = 0.002, one tail; Fig. 1*D*, top), thus suggesting a relationship between pupil size and alpha amplitude. Because of this relation showing a natural variability across subjects, we explored it from a more dynamic perspective (Extended Data Fig. 1-1).

### Occipital alpha power covaries with high-frequency components of pupil diameter

To study the temporal dynamics between pupillary diameter and alpha oscillation, we correlated pupillary signal and occipital alpha power during the minute of recording. Although the correlation value was low, it was highly significant (*r* = 0.111, *p* < 0.001; Pearson correlation). Individually the mean *r* value was low (*r* = 0.126, *p* = 0.0675); however, 14 of the 16 subjects obtained *p* values <0.05 (median *p* value across all subjects = 1.08 × 10^−6^). The signals exhibit a low correlation (*r*), but highly consistent (low *p*). Pupil dilation have been shown to modulate brain signals, so, as reported previously, we next explored the periods where pupil transits between dilation and constriction, peaks, and troughs (Joshi et al., 2016). Pupillary and EEG dynamics reflect more than a single underlying phenomenon on their own. If at some point they coincide in reflecting the same phenomenon, we expect to observe it when the changes are most dramatic. For this reason, we focus on the segments of pupillary dynamics where the most extreme state changes occur: the transition from dilation to contraction (peak) and from contraction to dilation (trough), events considered relevant in previous work on pupil diameter (Choe et al., 2016; Joshi et al., 2016; Kucewicz et al., 2018). To characterize potentially more complex dynamics of the interaction between alpha power and pupil diameter, we identified peaks and troughs in the high-frequency (0.2–1 Hz) pupil signal and segmented the EEG data accordingly from 1 s before to 1 s after the corresponding event (Fig. 2*A*). We found an average of 23.56 (±6.02 SD) peaks and 23.63 (±5.97 SD) troughs in a 1 min recording across subjects. We then decomposed the signal in the frequency domain to obtain a representation of the oscillatory components related to different pupil fluctuations. The power spectrum of peak and trough-centered EEG data showed a clear increase of power in the alpha band (Fig. 2*B*). This alpha activity was located in the occipital area and showed a significant increase during pupil signal peaks versus pupil signal troughs (Fig. 2*B*, inset; cluster-corrected permutation test, *p* = 0.0016). In line with the results of Figure 1*D*, these analyses showed a differential alpha modulation during peaks and troughs of the pupil signal.

Previous studies have shown distinctive modulation of neural activity during pupil dilation and contraction in mice (Reimer et al., 2014). This modulation can be masked when using a fixed window spectral decomposition. To determine whether such complex dynamics account for the results reported here, we performed a spectral decomposition in time, around pupil signal peak and trough occurrence. We found a modulation of the alpha power around peaks and troughs (Fig. 3*A*). By cross-correlating the pupillary diameter and alpha-power time courses, we observed an increase in power that reached its maximum at 310 ms before the pupil peak (Fig. 3*B*, left, Extended Data Fig. 3-1*A*, left). Subsequently, alpha power decreased to its lowest values at 300 ms after the peak. The dynamics of the alpha power related to the trough of the pupil signal showed the opposite effect, increasing from the lowest values at 348 ms before reaching pupil trough, and increasing until maximum values at 339 ms post-trough (Fig. 3*B*, right, Extended Data Fig. 3-1*A*, right). Notably, these couplings collapse when the pupil signal is swapped in time, thus disrupting the temporal relation with the EEG, and the same analysis is performed ((i.e. first ms of EEG with last ms of pupilometry), thereby demonstrating the criticality of the temporal order in the correlated signals (Extended Data Fig. 3-1*B*). The topographical distribution of the alpha activity showed a modulation in the posterior channels (Fig. 3*C*), and a significant difference between conditions in the window before and after pupil peaks and troughs (Fig. 3*C*; cluster corrected permutation test, *p* = 0.0004). In addition, as previously reported in rodents (Reimer et al., 2016; Pais-Roldán et al., 2020) and humans (DiNuzzo et al., 2019), the first derivative of pupil diameter showed a linear correlation with alpha power (*r*^2^ = 0.8695, *n* = 3000, *p* = 0, Pearson correlation analysis; Fig. 3*B*). Our results suggest the existence of a dynamic relationship between alpha power and pupil diameter over time.

Next, since the rise and fall dynamics of alpha power can vary in time, we have so far studied the processes as separate events. To expose the general relationship between pupil dynamics and EEG alpha power, we parsed the data in relation to the phase of the pupil signal. We found the highest amplitude of alpha activity at ∼1.83π (*p* = 5.4 × 10^−69^, Rayleigh’s test; Fig. 3*D*). In addition, we found a significant correlation between pupil phase and alpha power of *r*^2^ = 0.7337 (*p* = 0.00,065, using 5 ms bins). This result suggests a coupling between the pupil phase and alpha-band amplitude. In summary, we found that during gaze fixation, alpha power is tightly coupled to spontaneous fluctuations in pupil diameter, and that pupil diameter accounts for a substantial portion of alpha-power variance.

## Discussion

In this work, we studied the relationship between spontaneous pupil dynamics and EEG activity in humans during inactive wakefulness. As previously described, spontaneous activity is defined as not being linked to external events (Reimer et al., 2014; McGinley et al., 2015; Yüzgeç et al., 2018). We found a close relationship between alpha-band amplitude and the high-frequency dilation/contraction cycle of the pupil, with a maximum alpha amplitude of ∼310 ms before the peak of the pupil diameter signal and a minimum amplitude of ∼348 ms before the trough of the signal. This relationship between alpha amplitude and the phase of the pupil diameter at rest is important for understanding how ongoing brain activity relates to non-task-driven behavior under natural conditions. So far, most of the research on human pupillometry has focused on the dynamics surrounding visual stimulation. Those studies have shown a relationship between pupil diameter and different related cognitive processes, such as memory, attention, cognitive load, and emotional response (Mathôt, 2018). However, given the temporality of the tasks, it is difficult to correlate the pupillary response to ongoing characteristics of the EEG signal, as independent from the presented stimulus.

### Pupil diameter fluctuations

A characteristic feature of the pupil signal is its variation on different time scales. Slow pupillary modulations of high amplitude have been observed in rodents during explorative behavior, such as walking and whisking (Reimer et al., 2014; McGinley et al., 2015). However, during periods of inactive wakefulness, higher-frequency microdilations were observed in shorter time windows. These microdilations were embedded in the low-frequency, high-amplitude fluctuations during active periods. McGinley et al. (2015) report pupil fluctuations of ∼0.03 Hz for low-frequency microdilations and 0.3 Hz for high-frequency microdilations. However, pupil fluctuations can be induced up to frequencies >3 Hz by exposing the subjects to cyclic changes in luminance (Naber et al., 2013). We distinguished the following two components: one of a low frequency between 0.05 and 0.2 Hz, and a second one of a higher frequency between 0.2 and 1 Hz; here we focused only on the latter, which was clearly distinguishable in periods of inactive wakefulness. We did not detect the presence of spontaneous pupillary oscillations of higher frequency >1 Hz.

### Relationship between pupil diameter and brain states

A major part of previous work focuses on slow pupil diameter components, slow changes of pupil size, or the raw pupil signal. Here we added on to the limited evidence regarding the high-frequency pupil component and EEG activity. Our first results showed a linear relationship between high-frequency pupillary fluctuations and alpha amplitude. As these analyses do not consider temporal dynamics, we focused our analysis on peaks and troughs of the pupillometry signal and found a modulation of alpha amplitude around these events. Relationships between fluctuations in pupil diameter and brain activity have previously been described. McGinley et al. (2015) report that the rate of occurrence of sharp-wave ripples in the hippocampus has significant levels of coherence with pupil microdilations in mice. Furthermore, they found a close relationship between microdilations and transient depolarization of cortical neurons, which are apparent as a peak in coherence at ∼0.3 Hz, reflecting their 1-s-long time course. Consistent with our findings, Reimer et al. (2014) found that, during still wakefulness, variations can be observed in the amplitude of the low-frequency neural signal (2–10 Hz) in relation to the different phases of the fast pupil dilation–contraction cycle. In contrast with our results, they found lower amplitudes before the peak of pupil diameter and higher amplitudes just after the peak. These discrepancies could be largely because of the different nature of the signals reported in their study versus ours (i.e., membrane potentials in the former and electroencephalography in the latter). Their work also reported a wider frequency band, unlike our study, which focused on the occipital alpha band. In addition, they showed an enhanced response of visual neurons to preferred stimuli in the ascending phase of pupillary dilation, which coincides with the increase in alpha power reported by us. This could reflect tuning mechanisms by increasing spike-field coherence to prioritize salient stimuli (Jensen et al., 2012). Interestingly, they reported a close relationship between the activity of somatostatin-positive and vasoactive intestinal peptide-positive neurons and pupillary dynamics (Reimer et al., 2014), which allows hypothesizing about a mechanism derived from the activity of these neuron types and the alpha activity in cognitive processes, which are deployed in natural paradigms involving innate behavior. On the other hand, Reimer et al. (2016) report that the phasic activity of noradrenergic neurons is associated with high-frequency pupillary dynamics, with a maximum of neuronal activity taking place ∼1 s before the peak of the pupil diameter. This is in line with the role of LC-controlling noradrenergic inputs to the pupil circuit as well as the cortex. In addition, a close relationship is reported between the EEG alpha oscillation in the primary motor cortex and slow modulations of pupil diameter in mice during non-rapid eye movement sleep. This relation breaks down in the presence of cholinergic receptor antagonists, thus revealing a strong parasympathetic component in the control of slow changes in pupil diameter during this sleep stage (Yüzgeç et al., 2018). In humans, vagus nerve stimulation (VNS) has probed the involvement of the LC noradrenergic system in arousal. Sharon et al. (2021) showed that transcutaneous VNS produced pupil dilation and a decrease of alpha activity, which relates both signals to the action of the noradrenergic system. Although in line with our results and those of previous reports (Reimer et al., 2016), their experimental design (event related) does not allow speculation about the specific dynamics during spontaneous modulations that we show here. However, the sympathetic and parasympathetic interplay in the control of pupil diameter under different conditions and of the cholinergic and noradrenergic modulation of the cerebral cortex activity has been studied extensively, which opens the door for future research to investigate the relationship of the autonomic system with the amplitude of alpha oscillations (Liu et al., 2017; Gelbard-Sagiv et al., 2018; for review, see Schwalm and Jubal, 2017; Joshi and Gold, 2020). Finally, the present work shows a temporal relationship between the pupil size signal and alpha activity. A correlation between pupil diameter and the power of occipital alpha activity had previously been described (Ceh et al., 2020). Our work converges with these findings and deepens them by establishing a specific temporal relationship with the high-frequency component of pupillary dynamics, as indicated by the cross-correlation analysis. Further, we covered two arguable caveats of the previous work. First, we used shorter rest periods, thus avoiding the occurrence of pupillary hippus. Second, we recorded at the beginning of the session, hence the period studied is not influenced by the previous task.

### Mental states and performance

Some cognitive processes induce specific brain dynamics that are highly dependent on the ongoing state of the brain. Therefore, an individual’s performance in different tasks that require those cognitive processes might be affected by different brain states. As discussed earlier, we can assess these states directly by measuring brain activity or indirectly by monitoring pupil dynamics. Several studies conducted in mice, nonhuman primates, and human subjects show a significant association between mental states and performance in different types of stimulus-related tasks (Iriki et al., 1996; Wierda et al., 2012; McGinley et al., 2015; Van Den Brink et al., 2016; Gelbard-Sagiv et al., 2018; Podvalny et al., 2019). As these paradigms are historically based on the presentation of stimuli, the lack of the subject’s control over the timing of stimulus appearance generates the need to characterize the relationship between the baseline before the appearance of the stimulus and the performance of the subject. Ecological tasks are tasks that resemble the natural occurrence of sensory stimulus presentation. Under these conditions, the subject’s internal rhythms reflected by pupil diameter can be monitored, for example, to determine windows of greater attentional engagement (Gilzenrat et al., 2010). Although we do not measure task performance in this study, spontaneous fluctuations in pupil diameter allow us to determine different levels of arousal or attention during natural behavior.

### Alpha as attentional mechanism

Studying the oscillatory activity of the prestimulus EEG signal is important to demonstrate variations in perceptual processing to be related to changes in ongoing neuronal activity states. Several aspects, such as amplitude, frequency, and phase of the alpha oscillation, are associated with performance in different types of cognitive tasks. In double-flash discrimination tasks, for example, an inverse relationship was observed between the frequency of alpha before the stimulus and the discrimination threshold (Samaha and Postle, 2015). This has been proposed as an adaptive mechanism that reflects the level of activation of neuronal populations (Mierau et al., 2017). On the other hand, the amplitude of alpha increases ipsilaterally in relation to covert attentional deployment (Worden et al., 2000; Thut et al., 2006). Furthermore, this effect is localized to task-relevant areas (Worden et al., 2000). In a study involving monkeys, a tactile discrimination task was used, demonstrating that EEG alpha power in the sensorimotor cortex before stimulus presentation is inversely related to performance, which is further accompanied by a decrease in firing rate. In addition, there is a strong modulation of the firing rate based on the phase of the alpha oscillation, which reflects a general mechanism for establishing the state of cortical networks (Haegens et al., 2011). Similarly, using a double-flash (Milton and Pleydell-Pearce, 2016) or a masking task (Mathewson et al., 2009), an association was shown not only between amplitude and the individual’s reported perception but also between the prestimulus phase of the alpha oscillation and the individual’s reported perception. This influence was especially noticeable during periods of higher alpha amplitude, which is associated with periods of low attention (Mathewson et al., 2009; Jensen et al., 2012). These results indicate that the alpha phase reflects cyclical changes in neuronal excitability, which would underpin attentional deployment over time. The idea that alpha inhibits neural networks that are irrelevant to ongoing information processing is consistent with our findings. Our results show a peak of alpha amplitude directly before the peak in pupil diameter, which indicates a lower level of attention preceding the peak of pupil dilation. The alpha amplitude would then begin to increase and reach a maximum at 300 ms after the peak of dilation. Our results show a close relationship between high-frequency pupillary oscillation and alpha-band amplitude. Although we observed low-frequency pupillary fluctuations, the 1 min restriction of our recordings does not allow us to adequately resolve this component and establish its possible relationship with EEG activity. In part, the focus of our research was to study periods of inactive wakefulness when high-frequency oscillations were observed. However, longer periods of inactive wakefulness promote the appearance of hippus of the pupil (Bouma and Baghuis, 1971), which could increase with longer periods of time (Pomè et al., 2020). Studying subjects sustaining fixation for longer periods does not model physiological conditions, since it might appear tedious and rather unnatural for them. Consequently, we decided that 1 min observations would suffice to study pupillary oscillations within the range of 0.2–1 Hz. Future studies could use more engaging passive tasks, as for example free viewing of natural scenes, to study the relationship between EEG and slower oscillatory components of the pupil.

### Conclusion

In this study, we show a close relationship between spontaneous rapid pupil diameter fluctuations and the occipital alpha band in the human EEG signal during inactive wakefulness. Since evoked modulations of alpha amplitude have been associated with variations of attention or alertness, signal changes over time may indicate fluctuations in attentional load. Similarly, pupil fluctuations over time would indicate variations in the individual’s arousal level. The consistent temporal coincidence of these two measurements strongly suggests momentary changes of attentional state. The temporal correlation we describe should be confirmed for more ecological conditions, such as during active viewing tasks. Visual exploration through eye movements is understood as the displacement of attention over the visual field. However, the level of attention during visual exploration is variable, and, as such, there might be eye movements representing higher or lower attentional load, depending on intrinsic states. Our results can help to elucidate which eye movements carry the highest attentional relevance during natural behavior by monitoring the relationship of alpha amplitude and high-frequency pupil dynamics.

## Synthesis

Reviewing Editor: Nicholas J. Priebe, University of Texas at Austin Decisions are customarily a result of the Reviewing Editor and the peer reviewers coming together and discussing their recommendations until a consensus is reached. When revisions are invited, a fact-based synthesis statement explaining their decision and outlining what is needed to prepare a revision will be listed below. The following reviewer(s) agreed to reveal their identity: NONE. Note: If this manuscript was transferred from JNeurosci and a decision was made to accept the manuscript without peer review, a brief statement to this effect will instead be what is listed below.

SYNOPSIS

Two reviewers evaluated the manuscript “Alpha EEG Activity and Pupil Diameter Coupling During Inactive Wakefulness in Humans” and both reviewers agreed that the authors have established a link between alpha wave EEG activity and pupillometry in a very simple paradigm. While the reviewers recognized the importance of this work, there was consensus that the results of this study should be compared to other recent publications, which have explored the link between EEG and pupillometry. In addition, the authors explore only a limited frequency range of pupil changes, and it is not clear why that specific range was chosen. Finally, there was consensus that the data presentation could be more efficient, reducing the number of manuscript figures. Both reviewers provided detailed suggestions which you should find useful in revising your manuscript, if you decide to resubmit it to eNeuro or to another journal.

-----------

REVIEWER #1

ADVANCES THE FIELD

The authors reported a unique coupling feature between alphas power and pupil dynamics during gaze fixation in human subjects. The observation is robust and the manuscript is well written. I support the publication of this manuscript in eNeuron.

COMMENTS

This is a well-performed study on simultaneous EEG and pupillometry. A robust correlation feature was observed between alpha band power and pupil size changes. The manuscript is well written. I have a few minor concerns to be clarified:

1. It will be more informative to perform cross correlation analysis to present the lag time difference between two signals. And, it will be better to explain the neuronal processing underlying the 300-400ms delay.

2. The authors claimed a potential alpha power increase following pupil constriction (trough). A better discussion is needed to elucidate the underlying neuronal linkage.

3. The authors only focused on the higher frequency (>0.2Hz) fluctuation of pupil dynamics. The overall brain state discussion is based on the “high frequency” fluctuation. It will be better to specify this high frequency-specific state fluctuation in discussion and differentiate it from brain state fluctuation with lower frequency range.

4. At last, the reviewer is a bit puzzled why authors could not acquire the data with longer duration to analyze the low frequency signal fluctuation. (a better justification in method will be needed).

-----------

REVIEWER #2

ADVANCES THE FIELD

The relationship between spontaneous alpha and spontaneous pupil that the authors show is a clear and strong effect, obtained under minimal experimental constraints. Previous studies have looked at pupil and alpha activity but may not have focused on fully characterizing their baseline relationship during fixation conditions, so in that sense this work is novel. However, several of the figure panels contain somewhat redundant information, and it is not clear that the unique material presented is sufficient to be published as a stand-alone paper.

COMMENTS

This study provides a clear demonstration of the relationship between alpha and pupil collected under minimal experimental constraints. The simplicity of the experimental design and analysis is a strength, as is the clear and straightforward presentation of the data. However, the authors claim of novelty seem quite overstated at several points in the paper:

37: “...there is no evidence that links [alpha band activity] to pupil changes in time"

96: “there is no direct evidence in humans relating any aspect of

EEG signal to spontaneous pupil dynamics"

First of all, as they authors themselves point out, fixation is an effortful task, and thus not entirely “spontaneous”. There are quite a few papers that measure pupil and alpha EEG during tasks, and while most focus on more complex behavioral paradigms it doesn’t seem entirely fair to say there are no previous data of this type. Even the specific finding of correlation between pupil and alpha described here seems to be pretty visible in other studies that are looking at “spontaneous” activity, including some that are not cited here such as Ceh et al 2020 (Brain and Behavior; see high correlation between occipital alpha and pupil dilation in Figure 4).

Figure 1 is nicely presented. The division of the pupil fluctuations into low and high frequency is a good choice, and in subsequent figures the clarity of the relationship when it is presented locked to the high frequency pupil fluctuations is powerful. However, in my opinion this is really one result presented several different ways. Since the pupil is band-pass filtered, with essentially the same number of dilations and constrictions, the left and right panels in Figure 2C and Figure 3 are presenting basically the same data shifted by one half pupil cycle. Figure 4 summarizes it another way. All together Figures 2-4 show a similar effect in slightly different ways but easily could be condensed into a single figure.

Altogether I think additional data or analysis would help place this interesting and robust finding in the context of a more complete story that would be suitable for publication as a paper.
