# Supplementary figures and images for: Alpha EEG Activity and Pupil Diameter Coupling during Inactive Wakefulness in Humans
Source: eNeuro. 2022 Apr 13;9(2):ENEURO.0060-21.2022. doi: 10.1523/ENEURO.0060-21.2022 (PMC9014982; doi:10.1523/ENEURO.0060-21.2022)

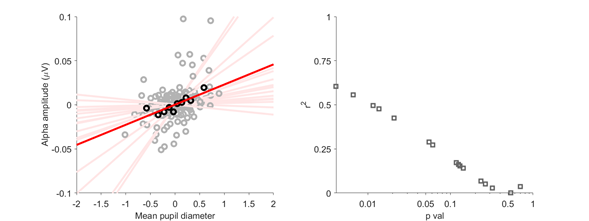

Supplement: Figure 1-1 — Individual correlation between pupil size and alpha amplitude. Left, Traces in light red correspond to each individual correlation between the pupil size deciles and the corresponding alpha amplitude at Oz location. Gray circles correspond to the averages of the decile used to calculate the correlations. Red line and black circles are the same averaged values shown in Figure 1D. Right, Black squares correspond to individual r2 and p value results from each individual correlation. Download Figure 1-1, TIF file. [file enu-eN-NWR-0060-21-s02.tif]

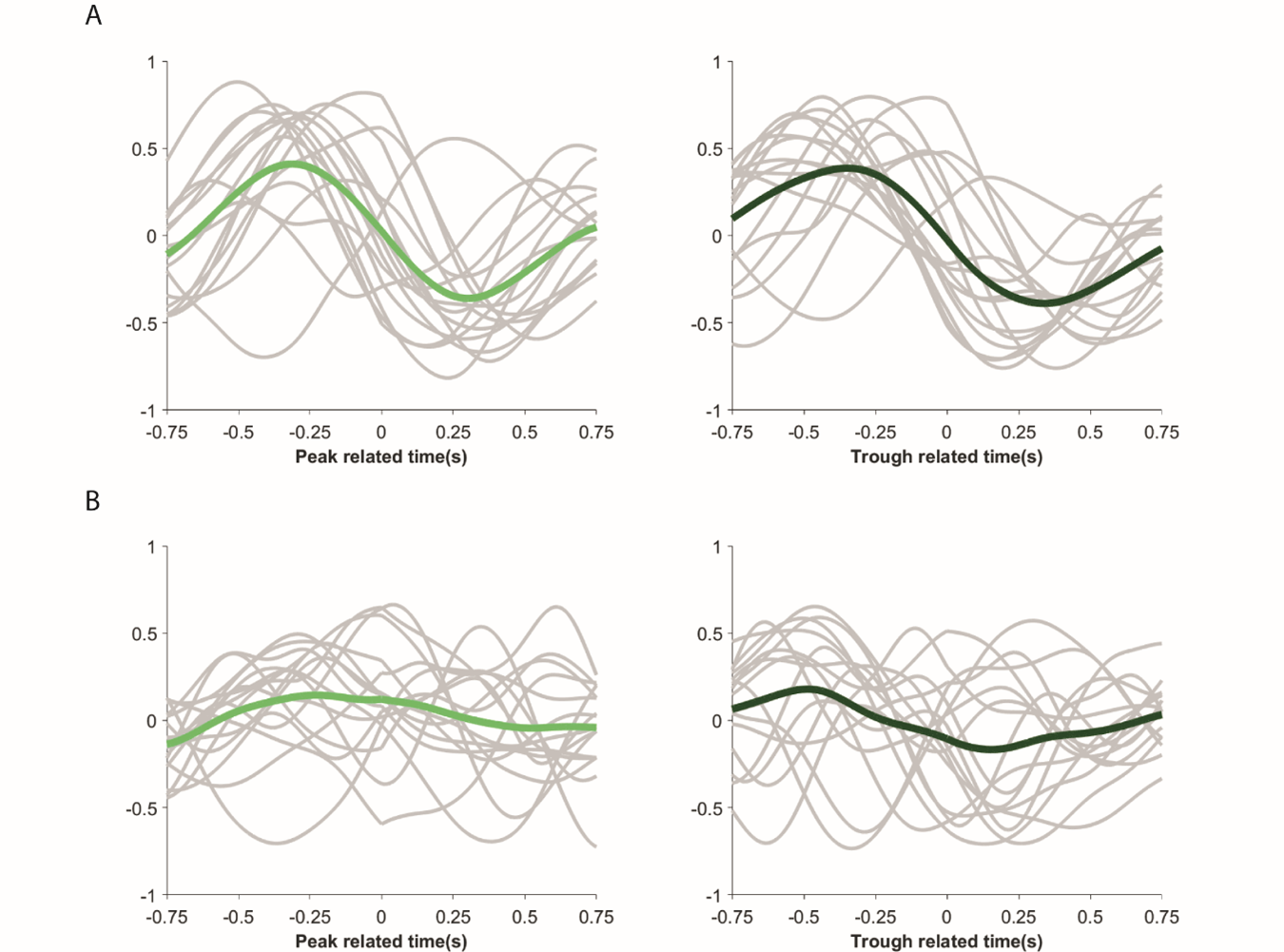

Supplement: Figure 3-1 — Normalized cross-correlation between pupil size and alpha power. A, Traces in gray correspond to each individual cross-correlation between the time course of the pupil size signal and the alpha power at occipital electrodes centered at peak pupil size. Green line represents the mean of all subjects (left). Same analysis as in left panel but centered at the trough of pupil size (right). B, Same as A, but for flipped EEG signal. Download Figure 3-1, TIF file. [file enu-eN-NWR-0060-21-s01.tif]
